# Brain Cancer Stem Cells: Current Status on Glioblastoma Multiforme

**DOI:** 10.3390/cancers3021777

**Published:** 2011-03-30

**Authors:** Sabrina Facchino, Mohamed Abdouh, Gilbert Bernier

**Affiliations:** 1 Developmental Biology Laboratory, Hopital Maisonneuve-Rosemont, 5415 Boul. l'Assomption, Montreal, H1T 2M4, Canada; E-Mails: sabrina.facchino@gmail.com (S.A.); abdouhm2009@gmail.com (M.A.); 2 Faculté de Médecine, Université de Montréal, Montréal, H3T 1J4, Canada

**Keywords:** polycomb, BMI1, cancer stem cell, glioma, glioblastoma multiforme, astrocyte, astrocytoma, radioresistance, CD133, prominin

## Abstract

Glioblastoma multiforme (GBM), an aggressive brain tumor of astrocytic/neural stem cell origin, represents one of the most incurable cancers. GBM tumors are highly heterogeneous. However, most tumors contain a subpopulation of cells that display neural stem cell characteristics *in vitro* and that can generate a new brain tumor upon transplantation in mice. Hence, previously identified molecular pathways regulating neural stem cell biology were found to represent the cornerstone of GBM stem cell self-renewal mechanism. GBM tumors are also notorious for their resistance to radiation therapy. Notably, GBM “cancer stem cells” were also found to be responsible for this radioresistance. Herein, we will analyze the data supporting or not the cancer stem cell model in GBM, overview the current knowledge regarding GBM stem cell self-renewal and radioresistance molecular mechanisms, and discuss the potential therapeutic application of these findings.

## 1. Introduction

Glioblastoma multiforme (GBM), a grade III or IV malignant astrocytoma as classified by the world health organization, is the most common and lethal primary brain tumor in adults [1]. Current therapies offered to patients include maximal exeresis, combined radio- and chemotherapy, and adjuvant chemotherapy [2,3]. However, even with these multiple interventions, the prognosis has improved minimally during the last decades. GBM is a heterogeneous brain tumor comprising a fraction of cells that resemble in their gene expression profile and phenotypic characteristics adult neural stem cells (NSCs) found in the brain. In all cases, GBM classification includes expression of the glial fibrillary acidic protein (GFAP) in cancer cells, a marker of astrocytes and NSCs. An overwhelming amount of literature favors the concept that brain tumor initiating cells in GBM arise from the transformation of cells residing in the subventricular zone (SVZ) of the cerebral cortex (the adult neural stem cell niche) rather than from parenchymal astrocytes—although the later possibility cannot be excluded for all GBM cases. Most importantly, several molecular pathways involved in normal NSCs self-renewal (which can be defined by the maintenance of stem cell proliferation and multiple differentiation capacities over time) are also implicated in GBM tumor growth. Therefore, understanding the mechanisms governing NSCs self-renewal could bring critical insight as to how GBM tumors grow and survive, thus providing potentially new therapeutic strategies against this highly lethal disease.

## 2. Neural Stem Cells

**Figure 1.**
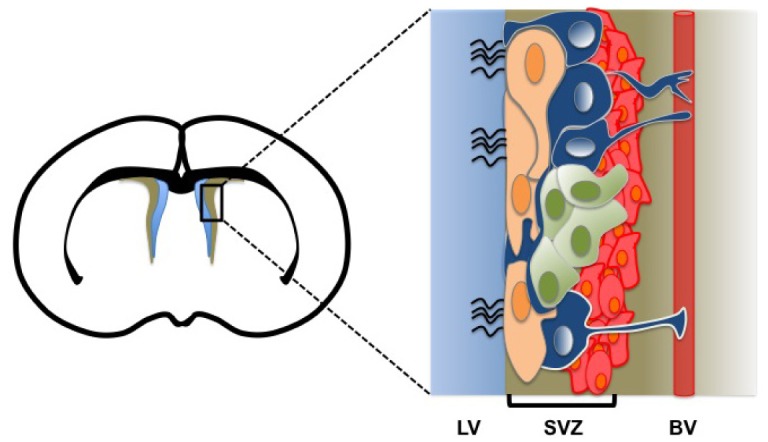
Scheme illustrating the mammalian neural stem cell niche. The left image represents a coronal view of the adult brain at the level of the lateral ventricule. The subventricular zone (SVZ; brown area) of the cerebral cortex is shown (black box). The enlarged box (right image) describes the architecture of the neural stem cell niche where resident type B cells (blue), C cells (green), A cells (red), and ciliary ependymal cells (peach) are shown. The lateral ventricle (LV) (blue area), the SVZ (brown area) and a blood vessel (BV; red rectangle) are also represented. Based on work from [13,14].

In the central nervous system, different NSCs and neural progenitor populations are found starting from early embryogenesis to adult stage. During embryonic brain development, neuroepithelial (NE) progenitors represent the most primitive NSCs. NE progenitors give rise to the first neurons and to basal progenitors (BPs). NE progenitors also produce an intermediate NSC population, the radial glia (RG) [4]. NE progenitors and RG cells can be distinguished by their morphology and expression of specific markers (Table 1). During fetal life, RG cells represent the principal cell type found in developing brain and serve as NSCs and support cells for migrating neurons [4-6]. RG cells display a more restricted differentiation potential compared to NE progenitors [6]. Like NE progenitors, RG cells give rise to BP cells, which primarily reside in the developing telencephalon. BP cells only produce neurons and represent the main neurogenic population during brain development [7-10]. NSCs persist after birth and are responsible for the maintenance of neurogenesis and gliogenesis in the developing and adult brain. Adult NSCs arise from the post-natal differentiation of RG cells [11,12]. Adult NSCs are found in precise regions of the brain, *i.e.*, the SVZ of the cerebral cortex and the subgranular zone (SGZ) of the dentate gyrus. NSCs reside in a specific microenvironment called the stem cell niche. At the ventricular surface, the niche displays a unique pinwheel structure composed of ependymal cells surrounding NSCs, and where NSCs retain a long basal process with blood vessels and a minute apical process with the ventricle [13] (Figure 1). This organization is presumably important for stem cells maintenance, neurogenic activity, and response to environmental cues. In the mouse SVZ, the stem cell niche contains at least four different cell populations: type A cells (neuroblasts), type B cells (quiescent NSCs), type C cells (transit-amplifying cells), and ependymal cells [14]. Type B cells express GFAP and hence are sometimes referred to as stem cell astrocytes (Table 1). Type B cells are responsible for the generation of type C cells, which have a high proliferation potential. Type C cells ultimately differentiate into neuroblasts that migrate to the olfactory bulb and generate interneurons [15-18]. A distinct and possibly more quiescent NSC population of ependymal cells may also exist in the mouse SVZ. These cells do not express GFAP but instead express the cell surface marker CD133/prominin-1 (Table 1). Like type B cells, CD133+/CD24- ependymal cells are able to self-renew and generate neurons, astrocytes, and oligodendrocytes [19]. The second source of neurogenesis in the adult brain is the SGZ. Radial astrocytes or type 1 progenitors within the SGZ represent the primary neuronal precursors. However, radial astrocytes do not directly produce neurons but instead produce an intermediate neurogenic cell population, the type D cell [20,21].

## 3. The Cell-of-Origin in GBM: Evidences from Animal Models

Because GBM tumors are histologically heterogeneous, containing cells expressing neural progenitor/stem cell, neuronal, and astroglial markers, it was proposed that these tumors could originate from the transformation of multipotent NSCs. Furthermore, the aggressive and invasive nature of this primarily adult tumor is suggestive of an embryonic or primitive origin. Holland *et al.* provided one of the first evidences that NSCs within the SVZ may be involved in gliomagenesis. By using viral-mediated transfer of oncogenes (RCAS/tv-a system) with an avian viral vector allowing expression of the target gene only in cells expressing the tv—a receptor—the authors were able to target specific cell populations. Two different transgenic mouse models have been engineered to express the tv—a receptor only in astrocytes (Gtv-a, GFAP expressing cells) or in neuroglial progenitors (Ntv-a, Nestin expressing cells). Infection of these animals with viral vectors expressing the constitutively active form of RAS and AKT resulted in the development of GBM-like tumor only in Nestin+ cells, thereby suggesting that type B or type C cells within the SVZ may represent the cell-of-origin [22,23]. However, additional loss of *p53* or *Ink4a/Arf* resulted in the formation of GBM-like tumors also in GFAP+ cells [24,25]. Whether these GFAP+ cells corresponded to astrocytes and/or type B cells is not known.

Notably, although *Ink4a/Arf*^−/−^ or *p53*^−/−^ mice do not develop spontaneous astrocytomas, immature astrocytes isolated from these animals are immortal and grow rapidly [26-30]. *P53*^−/−^ astrocytes also present chromosomal instability [30]. However, a recent study revealed that expression of a mutant form of p53 through Cre-mediated recombination in all GFAP+ cells, including their progeny, induced GBM-like tumor formation in mice only from cells located in the SVZ. This study further suggests that sole *p53* deficiency in cells located in the SVZ is sufficient to initiate the process of glioma formation [31]. These important findings are consistent with previous reports showing hyperplasia of the SVZ and NSCs overgrowth in *p53*^−/−^ mice [32,33]. Hyperplasia of the SVZ was also observed in mice injected with platelet derived growth factor-α (PDGF-α). The PDGF-α receptor is expressed by ∼80% of type B cells, but not by type-C cells [34]. It was also found that glioma-like tumors could develop from NG2+ progenitors located in the brain white matter of adult rats after ectopic expression of PDGF-α [35]. However, these GFAP^−^/NG2+/Olig2+ tumors showed hallmarks of oligodendrogliomas, a distinct tumor entity probably originating from the transformation of oligodendrocyte progenitors. Other investigators reported the development of GBM-like tumors arising from the SVZ following exposure of pregnant rats to N-nitrosourea (ENU), a highly potent mutagen [36,37]. Similar results were obtained using *p53*-deficient mice [32]. Importantly, these animals consistently developed glial tumors originating from GFAP+ or Nestin+ cells located in the SVZ. Recently, Alcantara *et al.* demonstrated using tamoxifen-inducible Nestin-CRE transgenic mice crossed with Nf1, p53, and Pten conditional mutants that only cells located in the SVZ develop into GBM-like tumors [38]. Likewise, viral-mediated recombination of Nf1, p53, and Pten in the SVZ or in non-neurogenic brain regions resulted in GBM-like tumor formation only from cells located in the SVZ [39]. These data suggest that only cells within the SVZ have the capacity to give rise to GBM-like tumors, while parenchymal brain cells, including astrocytes, cannot [38,39]. Interestingly, it was revealed that after simultaneous inactivation of *p53* and *Rb* in various brain regions of mice, again only cells located in the SVZ could generate tumors. In this case however, tumors displayed characteristics of primitive neuroepithelial tumors (a grade IV tumor), suggesting that specific genetic alterations may result in the transformation of adult NSCs into distinct brain tumor types [39]. Further evidence that GBM can arise from NSCs was shown using a transgenic mouse expressing an additional copy of the orphan nuclear receptor gene tailless (*Tlx*). In the adult mouse brain, Tlx is expressed exclusively in type-B cells. In this study, the authors demonstrated that Tlx acts as a key regulator of NSCs maintenance and expansion and also as a brain tumor-initiating cue for NSCs [40]. Thus, although neonatal mouse astrocytes can be easily converted into NSCs or transformed into malignant astrocytes *in vitro* [26-30,41], work using mouse models supports the hypothesis that the cell-of-origin in GBM is a NSC (or less likely a transit-amplifying cell) located in the lateral wall of the brain ventricles.

## 4. The Cancer Stem Cell Hypothesis

Because stem cells have an extensive proliferation capacity and can generate multiple different cell progenies, the heterogeneous composition of some tumor types makes normal stem cells attractive candidates as the cell-of-origin in these cancers. The cancer stem cells (CSCs) hypothesis stipulates that within a tumor, a small population of cells showing stem cell characteristics are at the origin of the tumor and responsible for tumor growth and maintenance [42,43]. By this principle, CSCs should be sufficient to reconstitute the original tumor upon transplantation in immune-deficient mice [44]. Thus, CSCs have been defined by analogy with normal stem cells.

The first evidence for the existence of CSCs arose from studies on acute myelogenous leukemia (AML). In these experiments, a subset of leukemic cells expressing cell surface markers normally present in hematopoietic stem cells were able to reconstitute AML in immune-deficient mice [45,46]. Thereafter, CSCs were also identified in solid tumors such as breast and brain cancers [47-51]. However, the CSCs hypothesis probably does not apply to all cancers. Notably, even in those where it was almost accepted, controversy remains. Recently, Quintana *et al.* contested that a small sub-population of cells within melanomas is responsible for tumor formation [52]. The authors concluded that the traditional xenograft assay using non-obese diabetic/severe combined immunodeficiency (NOD/SCID) mice grossly underestimates the number of tumor-initiating cells. They showed that only 0.1% of melanoma cells could generate secondary tumors in NOD/SCID mice, whereas the melanoma-initiating cell fraction was 25% in NOD/SCID/IL-2^−/−^ mice [52]. However, a similar study published later but using freshly isolated melanoma samples and primary cell lines cultured for limited numbers of passages strongly supported the idea that only a small population of CSCs is present in melanoma [53]. These two studies highlight how cell culture conditions and choice of a specific animal model can dramatically affect cancer cells phenotype and behavior. It also raised concerns about the use of cancer cell lines that have been maintained in culture for numerous passages or through serial xenotransplantion assays to study CSC biology.

## 5. Cancer Stem Cells in GBM

Evidences for the existence of CSCs in human GBM were first revealed by *in vitro* studies [49,50]. Dissociated cells from freshly isolated GBM tumors were able to form floating neurospheres when grown under NSC conditions, *i.e.*, in the absence of a coating matrix and serum, but with the addition of EGF, FGF2, and B27 supplement. Tumor neurospheres could be maintained through several passages and were able to produce neuronal and glial cells under differentiation conditions [49,50]. Notably, GBM cells grown under these conditions were found to express several NSC markers such as CD133/PROMININ-1, the intermediate filament NESTIN, the transcription factors SOX2 and BMI1, and the RNA binding protein MUSASHI [54-60] (see Table 1). In contrast, classical glioma cell lines maintained under serum-containing culture media do not reproduce the gene expression profile, NSC characteristics, and tumor phenotype of the tumor of origin [61]. Subsequently, the cell surface marker CD133 was used to isolate and characterize CSCs from GBM and medulloblastoma specimens [50,51,62]. Purified CD133+ cells, but not CD133- cells, could generate neurospheres in NSC culture media and produce glial cells and neurons in cell differentiation conditions. Other groups also confirmed that in contrast with CD133^−^ cells, CD133+ cells were able to induce brain tumors resembling the parental tumor following xenotransplant assays [63,64]. Therefore, these studies strongly suggested that brain tumor initiating cells in GBM were contained within the CD133+ cell fraction.

## 6. The CD133 Epitope

CD133 is a transmembrane glycoprotein expressed by hematopoietic stem cells, endothelial precursors and NSCs [65-67]. Even though the existence of CSCs within GBM tumor seems to be well accepted, its use as a universal marker to identify and isolate GBM stem cells remains controversial. An interesting study revealed that grafted CD133- GBM cells were able to generate brain tumors in nude rats. Furthermore, these tumors became positive for CD133 after serial orthotopic xenotransplant assays [68]. Thus, cell culture conditions apparently represent an important factor for the presence or not of the CD133 epitope in some GBM cell lines [69,70]. Mechanisms regulating expression and modifications of the CD133 epitope have been reviewed in detail elsewhere [71].

Likewise, several investigators have reported that over 40% of freshly isolated GBM specimens did not contained CD133+ cells, suggesting that CD133 is not an enrichment marker for CSCs in all GBM cases [72-76]. Rather, it was proposed that the stage-specific embryonic antigen-1 (SSEA1/CD15) may serve as a general marker for CSCs since SSEA1+ cells fulfilled the definition of CSCs and are present in all samples analyzed [76]. In support to their conclusions, the authors argued that expression of CD133 is not detected in type-B cells, in contrast with SSEA1 [77,78]. Notably, both CD133+ and SSEA1+ sorted GBM cell fractions were enriched for expression of the stem cell markers SOX2, BMI1, and EZH2 [76]. Taken together, this study strongly supports the cancer stem cells hypothesis in GBM but suggests that SSEA1 may represent a more universal cell surface marker for CSCs enrichment than the CD133 epitope.

In a more recent study, it was proposed that GBM tumors contain multiple distinct self-renewing populations that are organized into a lineage hierarchy [79]. In this report, the author analyzed 16 freshly isolated GBM tumors. All tumors that were positive for CD133, and both CD133+ and CD133-cells could generate expandable neurospheres with comparable efficiency. Likewise, both CD133+ and CD133- cells generated expandable neurospheres in clonal assays. Using 177 clones derived from 3 tumors, it was proposed that GBM tumors contain a range of self-renewing populations (type-I, -II and -III) where type-I CD133-/Nestin+ cells are the most immature. Although interesting, numerous aspects of this study must be taken into consideration. First, the near identical growth properties of CD133- and CD133+ cells in all 16 tumors is difficult to reconcile with numerous studies showing that the CD133+ fraction contains the self-renewing population in the vast majority of CD133+ GBM tumors [51,80,81]. Intracranial transplantation of freshly purified CD133- and CD133+ fractions would have provided valuable information on the cancer-initiating capability of these cells. Second, the derivation, expansion and maintenance for several passages of cell clones from a cancer cell line inherently open the possibility of a “natural selection process” for newly mutated variants having the greatest growth capability. This is important since most of the cell clones analyzed were derived from a single cell line already maintained through serial orthotopic grafts. Nevertheless, this study is potentially important because it could refute or change our perspective on the CSCs hypothesis in GBM. A reconciling point of view to the controversy surrounding the CD133 epitope may reside in the observation that a second NSC population may be present in the mouse SVZ: the CD133+/CD24-ependymal cells [19]. Hence, the CD133 epitope could represent the best selection marker for NSC enrichment in GBM tumors originating from the transformation of CD133+/CD24- adult ependymal NSCs. Ependymal cells are thought to derive from RG cells and to be at the origin of ependymomas. Ependymomas are mostly childhood brain tumors containing multipotent CD133+ CSCs that express the radial glia markers RC2, Nestin, CD133, and BLBP/FAB7 [64]. In contrast, the CD133 epitope may be irrelevant for NSC enrichment in tumors originating from type-B cells. In this latter case, the proposed lineage hierarchy model could possibly apply where transformed BLBP+/Nestin+/CD133-would give rise to both CD133+ and CD133-/DLX2+ populations having less aggressive phenotypes [79]. Although it is unknown whether CD133+ ependymal cells with NSC characteristics are present in the human SVZ, numerous CD133+/CD34-/CD45- cells having a normal karyotype and not expressing hTERT have been observed in GBM tumors after radiation therapy [82]. These cells presumably represent normal migrating NSCs attracted by the tumor lesion.

Notably, new experimental evidences suggest that stem cell populations within the mouse SVZ display a relatively high degree of plasticity. It was found that ependymal cells could give rise to astrocytes, and that astrocytes could give rise to ependymal cells. In this system, EphB2 and Notch signaling appear to actively inhibit the transition from ependymal cells to astrocytes [83]. Notably, although ependymal cells do not normally express SSEA1, ependymoma-derived neurospheres and ependymoma tumor samples are positive for SSEA1 [84]. At last, very recent findings suggest that adult NSCs located in the mouse SVZ express both GFAP+ and CD133+, in contrast with parenchymal astrocytes, SVZ astrocytes, ependymal cells, and type-C cells [85]. Taken together, these observations thus leave open the possibility that both CD133+/CD24- ependymal cells and GFAP+ type-B cells located in the SVZ are at the origin of GBM, and that both cell types can give rise to each other or share very similar characteristics, possibly depending on the micro-environmental context and type of transforming mutations.

## 7. Targeting Genetic Determinants of Stem Cell Identity in GBM

One prediction of the cancer stem cell hypothesis is that to self-renew, CSCs should depend on a similar network of stem cell regulatory factors as normal stem cells do. The prototypical stem cell factor Bmi1 has been extensively studied in stem cells from various organs, as well as in cancer cells [86]. In the mouse CNS, Bmi1 was shown to be required for NSCs survival and self-renewal through transcriptional repression of *p21^Cip1^*, *p19^Arf^* and *p16^Ink4a^* (the two later encoded by the *Ink4a/Arf* locus) [87,88]. The *Ink4a/Arf* locus is also the main target of Bmi1 proliferation-promoting activity in mouse embryonic fibroblasts and cerebellar granule cells [89].

**Figure 2.**
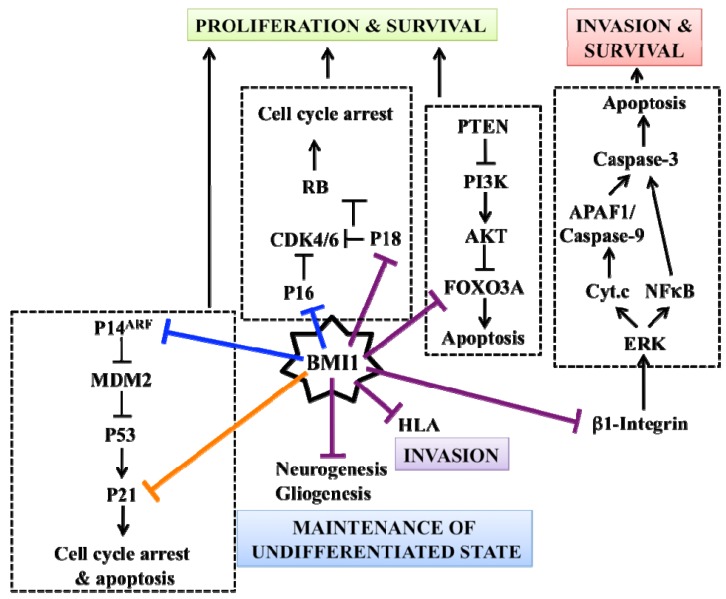
Oncogenic functions of BMI1 in Glioblastoma multiforme (GBM). BMI1 regulates multiple molecular pathways in order to maintain GBM cancer stem cells (CSCs) self-renewal capacity and promote tumor invasion. Previously identified BMI1 target genes are delineated by blue bars. Confirmed (orange bar) and potentially new target genes (purple bars) are also shown. Based on work from [70,145-147].

In a panel of 305 grade II, III and IV astrocytomas and oligodendrogliomas, BMI1 expression was detected in 302 tumors (99%) [90]. Work from the Van Lohuizen laboratory revealed that BMI1 is highly expressed in human GBM samples and that *Bmi1* deficiency reduces the invasiveness of malignant mouse astrocytes carrying a null mutation in the *Ink4a/Arf* locus [91]. Using *INK4A/ARF*-null human GBM specimens, we showed that BMI1 is enriched in CD133+ cells and required to sustain their self-renewal through prevention of CD133+ cells apoptosis and/or differentiation into neurons and astrocytes [70]. Out of 21 NOD/SCID mice transplanted with 1 × 10^5^ GBM cells, none developed brain tumors when *BMI1* was inactivated. Gene expression profile analysis revealed that one-way BMI1 promotes GBM cell survival and growth is through transcriptional repression of tumor-suppressor genes (such as *P18^INK4C^*) that attempt to compensate for *INK4A/ARF* deletion and PI3K/AKT hyper-activity (Figure 2). The robust expression of BMI1 in nearly all GBM samples analyzed and the extreme sensitivity of GBM cells to BMI1 inactivation further suggests that GBM stem cells have acquired an oncogenic addiction over BMI1 activity. Oncogenic addiction distinguishes CSCs from normal stem cells and can be viewed as a survival mechanism to overcome mutations affecting cancer cells viability [92,93]. This situation may render GBM stem cells more sensitive to BMI1 inhibition than normal stem cells present in the brain, and thus could be exploited in a therapeutic context. Other pathways involved in stem cell biology and also critical for GBM stem cells self-renewal, such as Notch, Sonic Hedgehog, Bone Morphogenic Protein, STAT3 and SOX2, have also been described [57,94-97]. Interestingly, it was also reported that the C-MYC oncogene is preferentially expressed in CD133+ GBM cells and required for CSCs survival and brain tumor engraftment in NOD/SCID mice [98]. C-MYC is the molecular cornerstone establishing the similarity between embryonic stem cells and cancer cells, and c-myc is negatively regulated by p53 and Pten in a murine model of GBM [99,100]. The identification of specific inhibitors against these factors, especially those involved in oncogenic addiction, may open new avenues to directly target CSCs for the treatment of GBM while preserving normal stem cell populations.

## 8. The Vascular Niche

Angiogenesis is considered as a crucial factor for the development and growth of GBM. Microvasculature proliferation can only be observed in high-grade glioma and this feature seems closely related to the aggressiveness and clinical recurrence of GBM [101-103]. Recent reports brought the biological basis underlying this phenomenon. Like normal NSCs [13,14,104], Nestin+/CD133+ self-renewing GBM stem cells home in perivascular niches that support them by removing metabolic by-products, and providing essential nutrients and maintenance cues [105,106]. Notably, endothelial cells can improve GBM stem cells survival and accelerate tumor initiation and progression [106]. On the other hand, GBM stem cells were reported to secrete Vascular Endothelial Growth Factor (VEGF) and Stromal-Derived Factor-1 (SDF-1), two potent angiogenic factors, thus promoting angiogenesis [105,107]. This paracrine relationship involving GBM stem cells and the neo-vasculature is particularly interesting since recent studies suggest beneficial effects of anti-angiogenic treatments with either the humanized VEGF-neutralizing antibody bevacizumab (Avastin) or the pan-VEGF receptor tyrosine kinase inhibitor cediranib (AZD2171) in recurrent high-grade glioma [108-110].

Paradoxically, GBM growth takes place in hypoxic microenvironment, which apparently helps supporting tumor neo-angiogenesis and malignancy. Hypoxia induces the expression of VEGF in GBM stem cells in a Hypoxia-inducible factors (HIF1 and HIF2)-controlled manner [111,112]. In parallel, hypoxia and HIFs increase the proportion of CD133+ GBM stem cells and promote their self-renewal [112,113,114,115]. Very recent works performed by two independent groups further revealed that the CD133+ cell population in GBM contains a subset of vascular endothelial cadherin (CD144)-positive cells showing CSCs characteristics and capable of *de novo* tumor vascularization through direct differentiation into endothelial cells [116, 117]. These findings suggest that a therapy targeting both CSCs and angiogenic factors would be required to inhibit GBM stem cells maintenance and tumor neo-vascularization.

## 9. Resistance to Chemotherapy and Radiotherapy

GBM is a highly aggressive tumor in part because of its ability to resist conventional chemotherapy and radiotherapy. Despite the fact that those therapies generally succeed in reducing the overall tumor size, relapse from an aggressive tumor and resistance are still the primary causes of poor survival rates. Recently, many studies have suggested that GBM CSCs might be the key element responsible for the resistance to therapies and tumor relapse. The primary chemotherapeutic molecule used to treat GBM is temozolomide (TMZ), an alkylating agent that O6-methylates the guanine. The DNA adducts generated are generally removed by the repair enzyme O6-methylguanine-DNA-methyltransferase (MGMT). GBM stem cells express high levels of MGMT and this may account for GBM resistance and recurrence following TMZ therapy [118]. Likewise, *MGMT* gene silencing (through *MGMT* promoter methylation) predicts a favorable outcome in patients exposed to alkylating chemotherapeutics and may help stratify or select GBM patients for clinical trials [119]. Normal stem cells also commonly express the ABC-transporters MDRT1 and BCRP, which are implicated in expelling toxic agents from cells [120]. Hence, increased expression of multidrug-resistance proteins promotes the efflux of chemotherapeutic agents by GBM stem cells, and targeting these transporters may improve the efficiency of chemotherapies [121,122].

GBM tumors are notorious for their radioresistance. Bao et *al.* first reported that the CD133+ stem cell population in GBM increased after radiation treatments *in vitro* when compared to the parental population, suggesting that CD133+ cells are more radioresistant than CD133-. It was suggested that CD133+ cells exhibit their high malignancy and resistance to radiation treatments through preferentially activation of the DNA damage response machinery, including the ataxia-telengectasia mutated (ATM) and Chk2 kinases. This mechanism was proposed to promote cell cycle arrest and efficient DNA repair in CD133+ cells, thus increasing overall cell survival [80]. The Chk1/Chk2 checkpoint kinases inhibitor debromohymenialdisine and poly (ADP-ribose) polymerase-1 (PARP1) inhibitors have been reported effective in rendering treated cells more vulnerable to radiations [80,123,124]. Notably, Tamura *et al.* observed by histological analysis of GBM specimens before and after patient's treatment that CD133+ cells survived to high radiation doses despite extensive damage to tumor blood vessels. The authors also noted a marked accumulation of CD133+ cells, particularly in remnant tumors within necrotic areas surrounding the irradiated zone, whereas these cells were infrequently detected in primary sections prior to treatment [125]. These results altogether suggested an enrichment of the CD133+ cell population after radiation therapy. However, it cannot be excluded that this observation in fact represents invasion of the tumor by normal NSCs expressing the CD133 epitope [82]. A better understanding of the basic mechanisms underlying GBM CSCs radioresistance could lead to the development of an efficient treatment against this cancer. We uncovered a novel role for BMI1 in the radioresistance capacity of GBM cells [81]. BMI1 was found to be redistributed on the chromatin upon gamma radiation treatments and to co-purify with DNA damage response proteins, including ATM and the histone variant γH2AX. BMI1 also preferentially co-purified with non-homologous end-joining (NHEJ) repair proteins in CD133+ GBM cells. Furthermore, BMI1 inactivation in GBM cells resulted in inefficient recruitment of the DNA damage response machinery, delayed DNA repair, and reduced cell viability [81]. BMI1 may thus represent a reliable target for the development of novel drugs against GBM, especially when combined with radiation therapy.

## 10. Conclusions

Tremendous experimental evidence exists in support of the CSC hypothesis in GBM. Transfering basic knowledge acquired in the field of NSC biology to GBM biology should lead to the development of novel therapies specifically targeting CSCs, thus possibly opening new avenues for brain cancer treatment. Future therapies against GBM targeting several CSC pathways simultaneously or exploiting the concept of oncogene addiction could dramatically improve the efficiency of actual chemotherapy and radiotherapy treatments while sparing normal stem cell populations.

## Figures and Tables

**Table 1.** Molecular markers associated with neural stem cells, progenitors, and cancer cells.

**Cell types**	**Markers**	**References**
*MOUSE EMBRYO*	Nestin, Sox1, Sox2, Pax6	[6,126-130]
Neuroepithelial progenitor		
*MOUSE FETUS*	Nestin, RC2, Sox2, Blbp, GLAST, Pax6, GFAP (human), CD133/prominin-1	[6,131-136]
Radial glia		
*ADULT MOUSE*		[6,19,34,137-144]
Type A cell (Neuroblast)	Doublecortin, filamin 1, L1 CAM	
Type B cell (neural stem cell)	GFAP, GLAST, Tlx, Nestin, Sox2, SSEA1, PDGF-□	
Type C cell (transit-amplifying cell)	Nestin, Dlx2, NG2	
Ependymal cell	CD133+/CD24+	
Ependymal ≪stem cell≫	CD133+/CD24-	
GBM cancer stem cell (human)	CD133, SSEA1, NESTIN, SOX2, BMI1, MUSASHI	[54-60,76]
